# Left lung cancer in a patient with congenital unilateral absence of the left pulmonary artery: a case report and literature review

**DOI:** 10.1186/s12957-020-1810-6

**Published:** 2020-02-07

**Authors:** Jing Wang, Xiaoqian Lu, Xiaobo Ding, Dian-bo Cao

**Affiliations:** Department of Radiology, The First Hospital of Jilin University, No.71 XinMin Street, Changchun, 130021 China

## Abstract

**Background:**

Unilateral absence of pulmonary artery (UAPA) is a rare congenital disease of pulmonary circulation, which is often accompanied by other cardiovascular anomalies. Infrequently, it may remain undiagnosed until adulthood. More rarely, it is to be found with lung cancer in the ipsilateral or contralateral lung simultaneously.

**Case presentation:**

A 56-year-old man with complaints of intermittent left chest pain for 2 months was referred to our hospital. Chest computed tomography(CT) revealed a cavitary lesion measuring 5.5 cm × 5.7 cm in the superior segment of the left lower lobe. Absence of left pulmonary artery and right-sided aortic arch were found on chest computed tomography angiography (CTA). The tumor was successfully removed via left pneumonectomy, and postoperative histopathology showed that the tumor was a squamous cell carcinoma (T2bN1). At a postoperative 24-month follow-up, the patient was free of disease and no evidence of recurrence or metastasis. Based on literature review, this is the ninth case of lung cancer in UAPA patients.

**Conclusions:**

Lung cancer and UAPA occurred ipsilaterally in 66.7% of these cases (6/9), including the present case. For those patients who occurred contralaterally, surgical treatment may be more challenging. CT and CTA could provide an accurate diagnosis for this disease entity. Identification and recognition of this rare and special disease entity may facilitate timely diagnosis and appropriate treatment.

## Background

Unilateral absence of pulmonary artery (UAPA) also means unilateral pulmonary artery agenesis. UAPA is a rare congenital anomaly and can remain asymptomatic for years, so the actual prevalence is difficult to identify. The estimated prevalence of isolated UAPA without other cardiac anomalies is around 1 in 200,000 individuals [1–3], which was calculated by cases per year in a certain medical center. It has no sex predilection and is often accompanied by other cardiovascular anomalies, such as Tetralogy of Fallot in pediatric patients. Presented symptoms may include dyspnea on exertion, recurrent pulmonary infections, hemoptysis, chest pain, and pleural effusion. Approximately 13 to 15% of patients with unilateral pulmonary artery agenesis remain asymptomatic and are diagnosed incidentally [1, 4, 5]. Digital subtraction angiography (DSA) has ever been the gold standard for establishing a diagnosis of pulmonary artery agenesis, and now been replaced by non-invasive methods like CTA. The occurrence of lung cancer in patients with UAPA is even rarer and experiences regarding surgical management are very limited. To our knowledge, only limited cases have been reported in the English literature. Therefore, we reported a case of unilateral absence of the left pulmonary artery together with ipsilateral lung cancer and made a literature review of eight cases on this rare clinical condition, focusing on imaging findings, pathology and treatment. Our aim is to identify the clinical features of UAPA accompanied by lung cancer more systemically and guide the future treatment for this entity.

## Case presentation

**Fig. 1:**
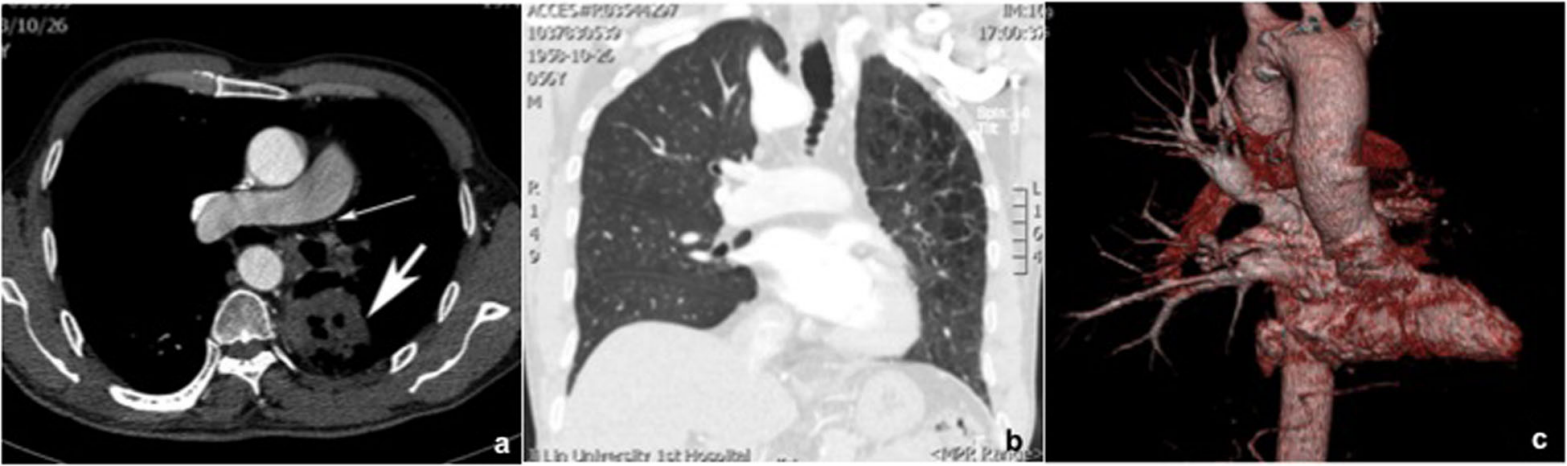
Preoperative chest CTA of a 56-year-old man showed unilateral absence of left pulmonary artery and pulmonary mass in left lower lobe. Axial contrast-enhanced CT showed a cavitary lesion measuring 5.5 cm × 5.7 cm in the superior segment of left lower lobe and absence of left pulmonary artery in its expected course (**a**). Coronal CT reconstructed image demonstrated the absence of left pulmonary artery and extensive emphysematous changes in the pulmonary parenchyma (**b**). Volume rendering CT revealed the absence of the left pulmonary arterial vasculature(**c**)

**Fig. 2:**
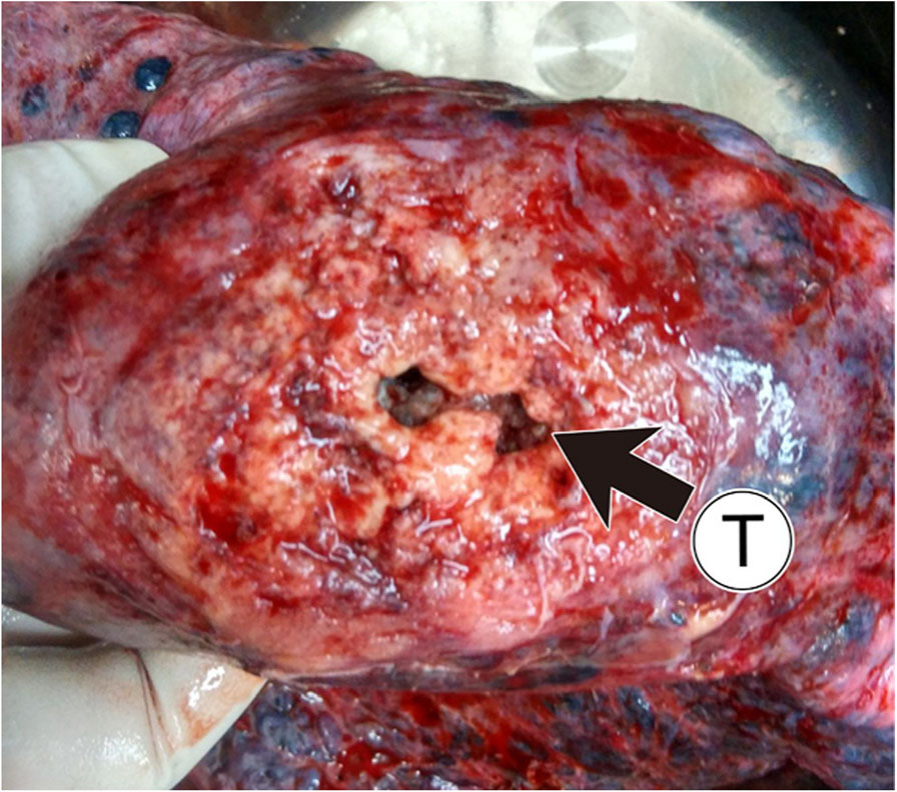
Gross photo of specimen illustrated a cavitary lesion with ill-demarcated margin(arrow). T, tumor

A 56-year-old man with intermittent left chest pain for 2 months was admitted to our hospital on June 19, 2015. He visited the local hospital owing to aggravated chest pain 6 days previously. Non-enhanced CT performed at his local hospital indicated a lesion in the lower lobe of the left lung, and his condition was not getting better even after a period of antibiotic administration. He denied a history of repeated pulmonary infection during childhood and had a 30-year heavy smoking history. Temperature was normal, and blood routine test was unremarkable. His serum tumor markers were all within the normal limits. Chest CTA in our hospital showed a cavitary lesion measuring 5.5 cm × 5.7 cm in the superior segment of the left lower lobe. Absence of left pulmonary artery and right-sided aortic arch were simultaneously found. Other findings included extensive emphysematous changes along with intrapulmonary infection in left lung and lymphadenopathy in the left hilum (Fig. 1). There were no other congenital cardiac anomalies to be observed except for right-sided aortic arch. Bronchoscopic examination was subsequently performed and squamous cell carcinoma on biopsy samples was highly suspected. Left pneumonectomy was optional to excise lung tumor and fibrotic lung under such circumstances. Intraoperatively, a neoplasm measuring 6.8 cm × 6.3 cm × 4.0 cm was seen in the superior segment of left lower lobe, adhering to the posterior chest wall. Many collateral vessels from the chest wall, mainly arising from the bronchial artery, made the operation difficult unexpectedly. Excised specimen revealed a cavitary lesion with ill-defined margin (Fig. 2). Histopathological examination was consistent with squamous cell carcinoma (T2bN1). At a postoperative 24-month follow-up, the patient was free of disease and no evidence of recurrence or metastasis.

## Literature review

We performed a PubMed literature search to identify cases of UAPA accompanied by lung cancer that had been reported between 1975 and 2019. Eight such cases were enrolled in eight papers. Table 1 shows the summary of the nine cases of UAPA accompanied by lung cancer including the present case.

**Table 1:** Summary of cases of unilateral absence of pulmonary artery accompanied by lung cancer

Case	Author/year	Age/sex	Symptoms	UAPA	Lung cancer Location	Radiology findings			Treatment	Surgical findings		Pathology findings	Postoperative complications
						Mass	UAPA	Other findings		Collateral vessels	Other findings		
1	Mancebo A /1975 [6]	49/F	Asymptomatic	Right	Right upper lobe	√	√	None	Mediastinoscopy, without thoracotomy or lung biopsy	None	Enlarged lymph nodes in mediastinum	Undifferentiated metastatic carcinoma of lymph nodes	None
2	Roman J/1995 [7]	54/M	Fever, chills, shortness of breath, chest pain, cough, brown sputum	Left	Left lower lobe	√	√	None	Left pneumonectomy	√	A hypoplastic left lung	Poorly differentiated adenocarcinoma	None
3	Ito M /2010 [8]	57/M	Asymptomatic *	Left	Right middle/lower lobe	√	√	None	Radiotherapy without surgery	Not available	Not available	Adenocarcinoma	None
4	Wozniak CJ /2011 [9]	67/F	Asymptomatic	Left	Left upper lobe	√	None	Right-sided aortic arch	Resection of the left upper lobe	√	Heavy pleural adhesions	Squamous cell carcinoma	None
5	George/2015 [10]	50/F	Episodes of recurrent hemoptysis, shortness of breath	Right	Right, three lobes	√ multiple	√	Emphysema	Right pneumonectomy and mediastinal lymph node dissection	√	Bullous changes, fibrous tissue at the hilum	Four adenocarcinomas two in situ adenocarcinomas.	Transient atrial fibrillation, treated medically
6	Yui Watanabe/2015 [11]	76/F	Asymptomatic	Right	Right lower lobe	√	√	None	Right lower lobe resection	√	A bloodless funicular structure	Not available	None
7	Zhang LZ/2016 [12]	60/F	Asymptomatic	Right	Left lower lobe	√	None	Interstitial changes in right lung	Left lower lobectomy with mediastinal lymph nodes dissection	Not mentioned	repeated decreases in SaO_2_ during one-lung ventilation	Adenocarcinoma	Persistent low SaO_2;_ bloody tracheal excretions; died^#^.
8	Kun Woo Kim/2018 [13]	56/M	Asymptomatic	Left	Right lower lobe	√	√	Hypertrophic bronchial arteries	Right lower lobectomy	Not mentioned	None	Adenocarcinoma	Dyspnea gradually improved.
9	Present case	56/M	Intermittent left chest pain	Left	Left lower lobe	√	√	Cavity, right-sided aortic arch emphysema; lymphadenopathy	Left pneumonectomy	√	None	Squamous cell carcinoma	None

*UAPA* unilateral absence of pulmonary artery; *F*: female; *M*: male; *SaO*_*2*_: arterial oxygen saturation

*Case 3 underwent two resecting operations for stomach and right lung adenocarcinoma five years ago

^#^Case 7 died of ventricular ectopia and cardiac arrest on the 2nd day postoperatively

The nine cases included five women and four men. Six of these patients were asymptomatic. One of them presented with fever, chills, shortness of breath, chest pain, and cough, and another complained of shortness of breath with episodes of recurrent hemoptysis. The chief complaint of our present case was intermittent chest pain.

The first case reported in 1975 was diagnosed on chest roentgenograms. The other eight of the nine patients underwent CT scanning, and pulmonary masses were all clearly visualized on CT scans. Seven cases of UAPA were diagnosed preoperatively, one of which with pulmonary angiogram, and six via CT scans. Lung cancer and pulmonary artery agenesis occurred on the same side in 6 cases. Among them, 3 cases were on the left side, and 3 cases were on the right side. The other 3 cases of lung cancer were found on the contralateral side of UAPA. Two cases were reported to have obvious emphysema and five cases with dilated collateral vessels. Right-sided aortic arch was reported in 2 cases. Our present case was the only one with cavity. Neither cavity nor calcification has been mentioned in previous cases.

The tumor was removed via pneumonectomy in 3 cases and via lobectomy in 4 cases. Another case underwent mediastinoscopy without thoracotomy or lung biopsy, and the other one received radiotherapy. The detailed surgical procedure was described exclusively in five cases, in which the extensive collateralization was found. Pathologic diagnosis was adenocarcinoma in five patients, squamous cell carcinoma in two, undifferentiated metastatic carcinoma of mediastinal lymph nodes in one and not documented in one.

Among the nine cases, 6 patients had no obvious postoperative complications, 1 had transient atrial fibrillation with a rapid ventricular response who was treated medically, and 1 had dyspnea and improved gradually. Postoperative death occurred in one case due to ventricular ectopia and cardiac arrest complications.

## Discussion

UAPA is a rare congenital anomaly. It is secondary to a failure in the connection of the sixth aortic arch with the pulmonary trunk [3]. It is often accompanied by other congenital cardiovascular malformations, such as tetralogy of Fallot in pediatric patients. Adult patients with UAPA may present with hemoptysis, exertional dyspnea, and recurrent pulmonary infection. However, about 15% of UAPA patients are asymptomatic owing to stable hemodynamics [1]. Fortunately, the sole right-sided aortic arch in our patient is not a hazard anomaly, which is probably responsible for delayed diagnosis of UAPA. The right-sided aortic arch is generally related to the agenesis of the left pulmonary artery instead of the right pulmonary artery. It may be ascribed to events occurring before or during the 5th or 6th gestation week which affect the formation of the left pulmonary artery and the left ductus arteriosus [7, 14]. As described in most patients with UAPA, our patient remained asymptomatic and undiagnosed until incidentally detected on imaging studies because of certain unrelated symptoms, such as progressively deteriorated chest pain caused by lung cancer.

**Fig. 3:**
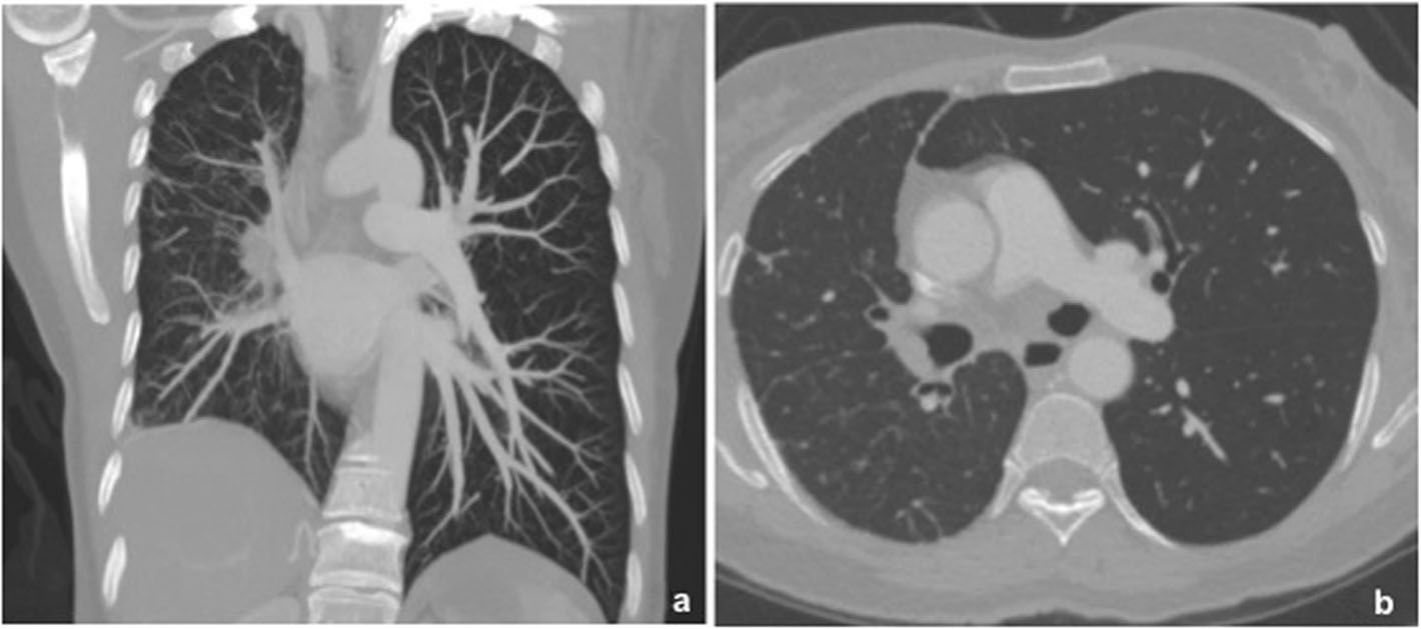
Chest CT of a 48-year-old woman demonstrated right pulmonary artery agenesis. Coronary CT reconstructed image showed smaller hemithorax, elevation of the hemidiaphragm and paucity of lung vascular markings on the right lung suggesting right pulmonary artery agenesis (**a**). Axial contrast-enhanced CT revealed the absence of right pulmonary artery within 2 cm of its expected origin from the main pulmonary artery (**b**)

**Fig. 4:**
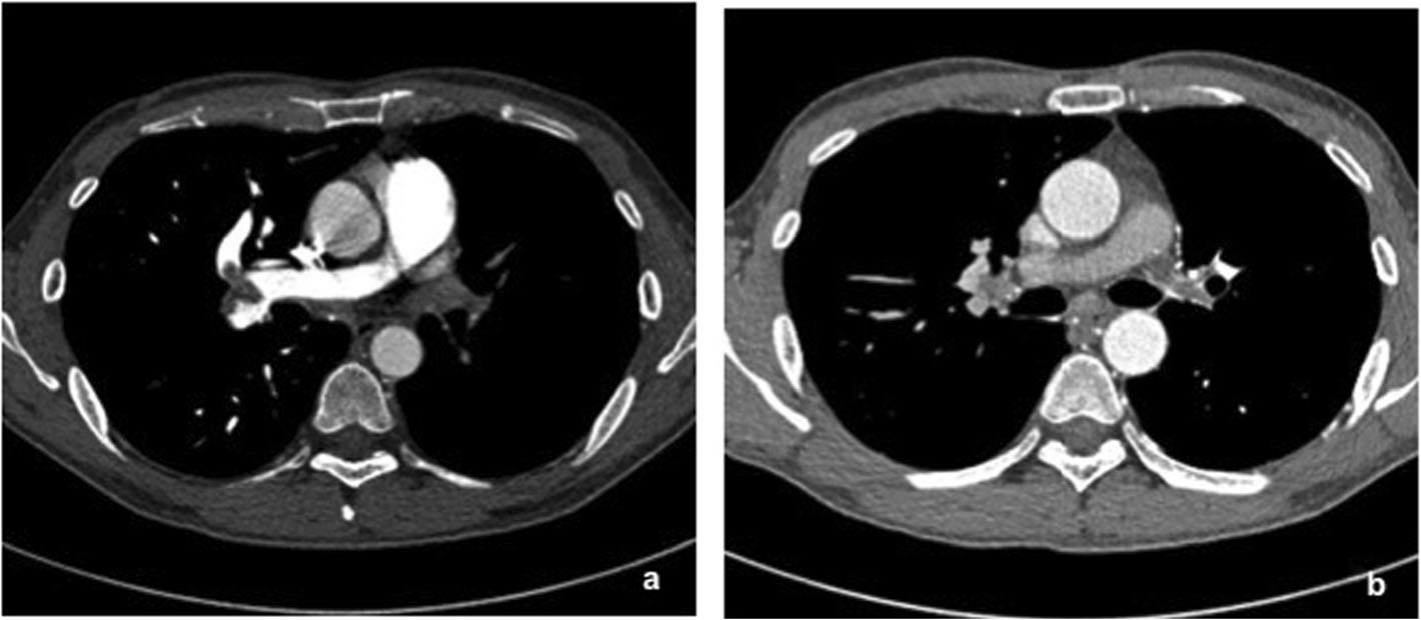
Thromboembolic disease of pulmonary artery on dynamic contrast-enhanced CT in a 44-year-old man. Axial CT revealed eccentric filling defects in the distal right pulmonary artery and its main branches suggesting acute pulmonary embolism(**a**). On the upper level, delayed CT revealed narrowing left pulmonary artery stump caused by chronic thromboembolic disease (**b**)

Chest X-ray, ventilation-perfusion scans, ultrasonography, CTA, and DSA may be helpful in establishing the diagnosis of UAPA. Chest radiographic findings include ipsilateral cardiac and mediastinal displacement, absent pulmonary arterial shadow, smaller hemithorax, elevation of the hemidiaphragm, and paucity of lung vascular markings on the affected side. There may be hyperinflation and herniation of the contralateral lung across the midline [15, 16]. A ventilation-perfusion scan shows the ipsilateral absence of perfusion with intact ventilation. Contrast-enhanced CT plays a vital role in diagnosing this congenital anomaly, although conventional DSA is ever the golden standard. CTA can definitely delineate the absence of the affected pulmonary artery and extensive systemic collateral circulation arising from the bronchial, intercostal, subclavian, subdiaphragmatic or segmental vessels [4, 12]. Meanwhile, CTA is also essential to reveal complicated cardiovascular malformations and intrapulmonary lesions. In our case, CTA provided the exact diagnosis and detailed information for surgery. UAPA may occasionally be mistaken for unilateral chronic thromboembolic disease because both may present with the absence of ipsilateral lung artery shadow and collateral vessels. Combining with clinically relevant data, some imaging findings could give some clues in differentiating two disease entities. Mediastinal displacement, smaller hemithorax, lung hypoplasia associated with obliterative bronchiolitis, and multiple cysts are strongly suggestive of UAPA. Besides, the absence of pulmonary artery usually terminates within 2 cm of its expected origin from the main pulmonary artery (Fig. 3). By contrast, abrupt vessel narrowing or occlusion, eccentric filling defects with partial intraluminal extension, and no displacement of mediastinum are more favorable for chronic thromboembolic disease (Fig. 4).

UAPA accompanied with lung cancer is an extremely rare condition. To our knowledge, there are only 8 cases documented in the English literature since December 1975 (Table 1). The ninth one is the present case. We found 695 cases of congenital UAPA in the literature [17]. The lung cancer incidence in UAPA patients is higher than that of the overall population (9/695 versus 23-50/100000 in China in 2014 [18]). Based on our patient and literature review, lung cancer is more liable to occur in the ipsilateral side of UAPA (6 in 9 cases). This possible association between the two entities deserves further study. Mechanism about the coexistence of UAPA and lung cancer remains unclear. We speculate the possibility that structural developmental abnormalities in the hypoperfused lung predispose the patients to the development of cancer, irregardless of the histopathological type. George Makdisi also points out a possible mechanism of the occurrence of lung cancer with UAPA that chronic hypoxia may cause DNA damages. Chronic hypoxia may result in the release of reactive oxygen species (ROS), induction of hypoxia-inducible factor-1(HIF-1) and p53, and induction of cell proliferation, leading to the occurrence of cancer [10, 19].

To date, no consensus was reached on the management of patients with UAPA. Pneumonectomy and surgical revascularization, selective embolization of systemic arteries, and pharmacological treatment for pulmonary hypertension are optional treatments for UAPA [5]. Surgical treatment for UAPA is considered for patients in whom associated anomalies are present or for those who are symptomatic. Surgical strategies may include revascularization for pulmonary hypertension or cardiac failure and pneumonectomy or lobectomy for hemoptysis or recurrent lung infection. The prognosis of UAPA depends on the severity of the complicating cardiovascular anomalies or pulmonary hypertension, or both [3, 15]. Early surgical intervention for the reestablishment of pulmonary blood flow may potentially allow the affected lung to develop and prevent morbidity and mortality in children [20].

For those patients with UAPA and ipsilateral lung cancer, surgical treatment would be a safe option, and pneumonectomy of the affected lung is strongly recommended. However, in patients with UAPA and contralateral lung cancer, meticulous consideration should be given for surgical treatment. Limited resection including segmentectomy and wedge resection may be feasible [8]. Kim [13] reported such a contralateral case undergoing lobectomy successfully, whose performance status and all preoperative tests were within normal limits. It was suggested to prepare an extracorporeal membranous oxygenator preoperatively for coping with potential complex situations [13]. The only reported case of absent right pulmonary artery and left lung cancer died from postoperative complication, alerting us that full awareness and evaluation of possible anomalies including right heart function and pulmonary pressure should be necessary before surgery [12]. For patients with marginal cardiopulmonary function, more preoperative evaluation including pulmonary artery occlusion test or pulmonary perfusion scan may be valuable [21]. Postoperatively, it is extremely important to detect and monitor pulmonary hypertension or other potential critical situations. Irregardless of the conditions above, familiarities with extensive arterial collaterals in the pleural space are paramount for effective control of intraoperative bleeding. Embolizing collateral vessels before surgery would be an option to reduce intraoperative bleeding in those patients with extensive large collaterals.

## Conclusions

We described a case of squamous cell carcinoma in the left lower lobe, who was diagnosed left UAPA with right-sided aortic arch preoperatively. There is no direct evidence for the correlation between UAPA and lung cancer development although limited cases tend to occur in the ipsilateral side of UAPA. CTA provides direct evidence of UAPA, secondary pulmonary changes, and suspected lung cancer. Identification and recognition of this rare and special disease entity may facilitate the exact diagnosis and appropriate treatment.

## Data Availability

We declared that materials described in the manuscript, including all relevant raw data, will be freely available to any scientist wishing to use them for non-commercial purposes, without breaching participant confidentiality.

## Abbreviations

CT: Computed tomography CTA Computed tomography angiography DSA Digital subtraction angiography HIF-1 Hypoxia-inducible factor-1 ROS Reactive oxygen species UAPA Unilateral absence of pulmonary artery
